# Emission, Transport, and Deposition of visible Plastics in an Estuary and the Baltic Sea—a Monitoring and Modeling Approach

**DOI:** 10.1007/s00267-021-01534-2

**Published:** 2021-09-10

**Authors:** Gerald Schernewski, Hagen Radtke, Esther Robbe, Mirco Haseler, Rahel Hauk, Lisa Meyer, Sarah Piehl, Joana Riedel, Matthias Labrenz

**Affiliations:** 1 Leibniz Institute for Baltic Sea Research, Rostock, Germany; 2 Marine Research Institute, Klaipėda University, Klaipėda, Lithuania; 3 Hydrology and Quantitative Water Management Group, Wageningen University, Wageningen, The Netherlands

**Keywords:** Stormwater, Sanitary sewer overflow, Pollution, Monitoring, Microplastic, Cigarette butts

## Abstract

was to assess whether a comprehensive approach linking existing knowledge with monitoring and modeling can provide an improved insight into coastal and marine plastics pollution. We focused on large micro- and mesoplastic (1–25 mm) and selected macroplastic items. Emission calculations, samplings in the Warnow river and estuary (water body and bottom sediments) and a flood accumulation zone monitoring served as basis for model simulations on transport and behavior in the entire Baltic Sea. Considered were the most important pathways, sewage overflow and stormwater. The coastline monitoring together with calculations allowed estimating plastics emissions for Rostock city and the Warnow catchment. Average concentrations at the Warnow river mouth were 0.016 particles/m³ and in the estuary 0.14 particles/m³ (300 µm net). The estuary and nearby Baltic Sea beaches were hot-spots for plastic accumulation with 6–31 particles/m². With increasing distance from the estuary, the concentrations dropped to 0.3 particles/m². This spatial pattern, the plastic pollution gradients and the observed annual accumulation values were consistent with the model results. Indicator items for sewer overflow and stormwater emissions exist, but were only found at low numbers in the environment. The considered visible plastics alone can hardly serve as indicator for microplastic pollution (<1 mm). The use of up-scaled emission data as input for Baltic Sea model simulations provided information on large scale emission, transport and deposition patterns of visible plastics. The results underline the importance of plastic retention in rivers and estuaries.

## Introduction

The Baltic Sea is one of the largest brackish water bodies in the world and, despite all efforts, a pollution hot-spot (HELCOM 2018a). The Baltic Sea catchment is about four times larger than its surface area (420,000 km^2^) and it is inhabited by about 85 million people living in nine countries. Due to the humid climate, the mean annual riverine runoff to the Baltic Sea is with 14,425 m³/s (HELCOM 2018b) comparably high. Since human activities are the source of microplastics, wastewater is considered as a major emission pathway (e.g., Mintenig et al. 2016; Ziajahromi et al. 2016; Kay et al. 2018; Prata 2018). In the Baltic Sea region, the vast majority of sewage water undergoes a treatment. Depending on the quality of wastewater treatment, Baresel and Olshammar (2019) assume a microplastics retention between 85 and 98%. This efficient sewage treatment is one explanation for the relatively low estimated microplastic emissions to the Baltic Sea (Siegfried et al., 2017). Plastics above 1 mm in size are practically fully kept back during waste water treatment. On the other hand, microplastic emissions with sewer overflow water, e.g., after heavy rains, seem to be an underestimated pathway. Sewer overflow water consists of stormwater and untreated wastewater. In the Baltic, overflow events happen rarely. Despite that, Baresel and Olshammar (2019) conclude that the annual discharge of microplastics from sewer overflows can be in the same magnitude as the emissions with all treated wastewater. Therefore, for plastics above 1 mm in size, large micro-, meso-, and some macroplastics, sewer overflows and stormwater are very likely by far the most important emission pathway in the Baltic region.

Existing calculations of microplastic emissions to the Baltic Sea by Siegfried et al. (2017) or Bollmann et al. (2019) are conceptual, utilize only limited and aggregated data, and possess a very high uncertainty. Further, these values differ strongly from each other and they focus on mass calculations. Baresel and Olshammar (2019) provide comprehensive data on urban waste water and its treatment, covering the entire Baltic Sea region. Schernewski et al. (2020) use this data to estimate the annual emission of different microplastics size-fractions and plastic polymers to the entire Baltic Sea. This approach provides detailed spatial emission patterns, taking into account all relevant cities and rivers. Additionally, the seasonality of sewage overflow including stormwater is assessed. This data is used to carry out 3D-model simulations on transport, behavior and deposition in the Baltic Sea environment. In a follow-up model study, Schernewski et al. (2021) expand the approach to a wider spectrum of microplastic polymers, covering most plastics emitted to the environment. The simulations suggest average annual microplastic concentrations for various sea areas that correspond to the rare existing data (Setälä et al. 2016; Tamminga et al. 2018).

Model simulations show high concentrations near river mouths as well as in enclosed and semi-enclosed coastal waters (Schernewski et al. 2020). These findings are supported by literature, as well: Gewert et al. (2017) found a nearly ten times higher abundance of plastics in surface water near central Stockholm than in offshore areas. Yonkos et al. (2014) reported the highest microplastics concentrations near densely populated areas of Chesapeake Bay and comparable results exist for other estuaries and lagoons (Gray et al. 2018; McEachern et al. 2019; Song et al. 2015; Vermeiren et al. 2016; Vianello et al. 2013). However, the existing field data from surface waters is not sufficient to validate model results from the Baltic Sea. Due to high costs for microplastics sampling and analyzing in the sea, it is not likely that sufficient data will be available in the near future. Therefore, new approaches are required to get a reliable, validated, spatio-temporal pattern of marine (micro)plastics pollution.

The simulations of Schernewski et al. (2020) indicate, that microplastic fractions have an average residence time of only about 14 days in the Baltic Sea. The model approach assumes an efficient beaching of particles nearshore, in the wave zone. According to these results, shorelines serve as major sink and trap for microplastics. As a consequence, microplastics sampling could focus on the tidal accumulation zone of beaches for obtaining a better insight into marine microplastics pollution. Sampling microplastics at beaches is complicated, especially in micro-tidal seas such as the Baltic Sea, standardized methods still do not exist and the sample preparation and analysis is expensive. Therefore, we follow an alternative approach and focus on larger plastic fractions for which suitable methods exist (e.g., Haseler et al. 2019). The idea is that coastal pollution pattern of large micro- and mesoplastic as well as specific litter items may serve as indicator for the pollution of smaller microplastic fractions.

Overall aim of our study is to assess, whether a comprehensive approach linking existing knowledge with monitoring and modeling can provide an improved insight into the plastics pollution problem in the coastal and marine environment. Detailed objectives are to (a) combine field data with literature data and calculate the annual emissions resulting from urban sources, (b) extrapolate the results to receive emission data for wastewater related urban pathways in the entire Baltic Sea region as model input, c) perform 3D-model simulations on transport, behavior and deposition of larger micro- and mesoplastics as well as cotton sticks and cigarette butts in the Baltic Sea environment, (d) validate the model results with data from beach tidal zone monitoring at the southern Baltic Sea, (e) assess the role of plastic retention in river basins and the estuary and f) discuss the role of plastic items and meso-plastics as indicator for microplastic in the aquatic environment.

The work shall provide a better insight into emission, transport and behavior of meso- and large micro-plastics in the marine environment. This shall (a) enable steps towards an improved, cost-effective monitoring considering plastic size fractions as well as sampling locations and frequency; (b) support decisions on mitigation measures and (c) point out gaps in our understanding of the plastic problem in the coastal and marine environment.

## Study Site and Methods

### Study Site

The Baltic Sea has a surface area of about 420,000 km^2^. With an average depth of 55 m it is relatively shallow and has a long coastline of about 8,000 km. Strong salinity gradients maintain an estuarine circulation and a water exchange with the North Sea. As a sheltered brackish sea, the tidal range is below 0.2 m and tidal currents play only a minor role for transport processes. All our field studies focus on the German southern Baltic Sea coast and especially the Warnow Estuary. The southern Baltic Sea coast is formed by dunes, soft cliffs and long sandy beaches and tourism is a major economic factor. Several estuaries with larger cities form the economic centers of the German Baltic Sea region, such as the Flensburger Förde, the Kieler Förde, the Trave Estuary, the Oder Estuary, or the Warnow Estuary.

**Fig. 1:**
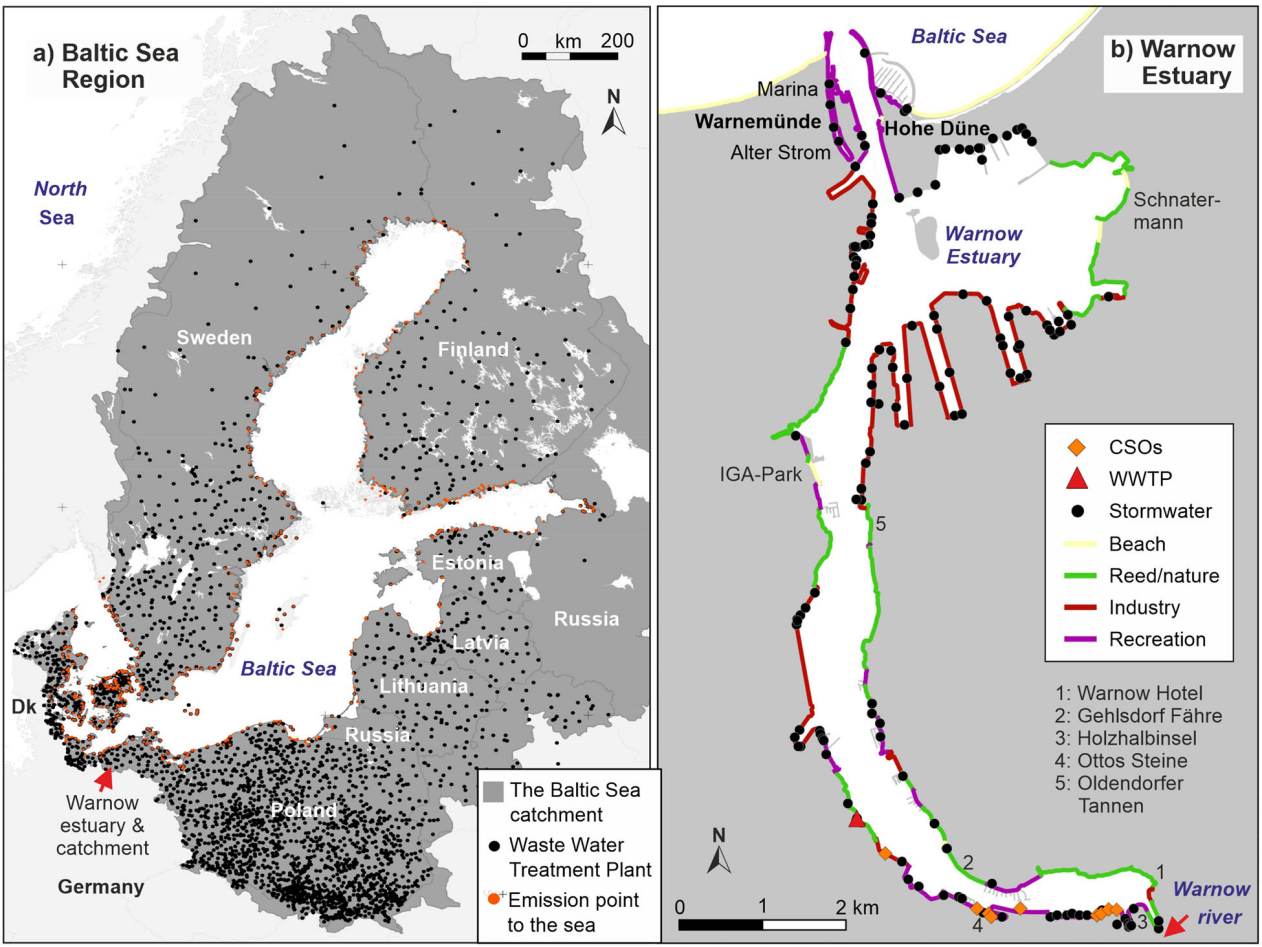
**a** The Baltic Sea Region, its catchment and the location of Waste Water Treatment Plants (WWTPs) as well as emission points to the Baltic Sea; **b** The Warnow Estuary (surrounded by the city of Rostock) with coastline structures and potential plastic emission points: WWTPs, combined sewer overflow (CSO) and stormwater (rain water discharge) outlets

The Warnow Estuary, surrounded by the city of Rostock (about 209,000 inhabitants), can be regarded as a representative southern Baltic estuary (Fig. 1). 74% of the inner shoreline are occupied by commercial and sport boat harbors, shipbuilding industry and hard structures protecting urban areas. The remaining shoreline is covered by reed belts, artificial stony shore and few beaches. The estuary has a length of about 14 km, a surface area of 12 km^2^ and an average depth of 5.6 m. The entire estuary is used for shipping and the channel has a maximum depth of 4 m in the city harbor in the south and 16 m between Baltic Sea and the industrial harbor in the north. Near the Warnow river entrance, the bottom salinity in the estuary is below 5 PSU compared to 18 PSU close to the Baltic Sea. These gradients are sufficient to maintain an estuarine circulation. The transport of surface water towards the Baltic Sea is additionally supported by the dominating western to south-western winds. The average water exchange time of the estuary is about 30 days (Lange et al. 2020). Twenty four small tributaries and the Warnow River discharge into the estuary.

The nearly 150 km long Warnow River drains a catchment with a population of about 200.000 inhabitants and a surface area of 3280 km^2^. It enters the estuary in the south and has an average annual discharge of 16.5 m^3^/s.

**Fig. 2:**
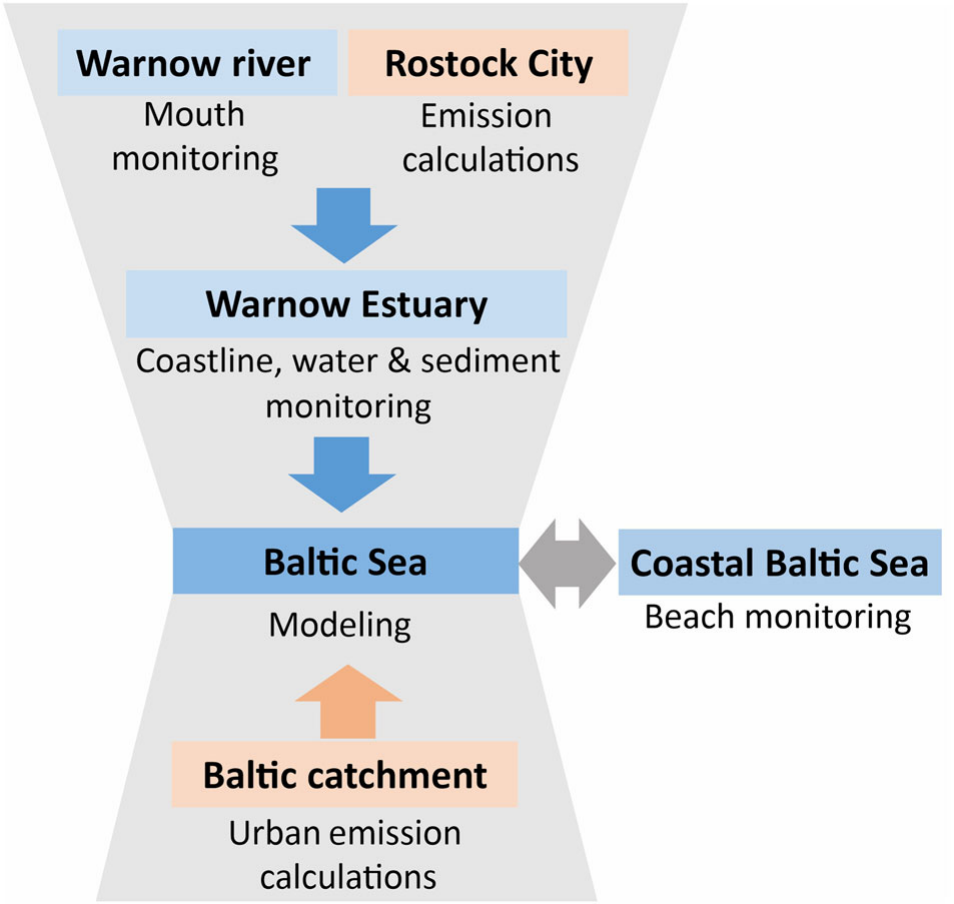
The conceptual approach

We focused on the large micro- and mesoplastic size class (1–25 mm) and in the following call this class “visible plastics”. In addition, we considered selected macroplastic items, which are supposed to provide an insight into the relevance of single emission pathways, and are therefore called “indicator items”. The monitoring at the Warnow river mouth together with emission calculations from Rostock City area provided information on the total emissions from urban sources to the Warnow Estuary (Fig. 2). Compared to agricultural sources, urban sources can be considered as major pathway in the Warnow river, as well (Tagg, pers. com.). This complied emissions data was validated by a coastline, waterbody and sediment monitoring in the estuary. Since each single approach was too weak to provide a full picture of the role of visible plastics and indicator items in the estuary, all approaches were combined. The resulting information on visible plastics and indicator item emissions served as input for the model. The model covers the entire Baltic Sea and requires emission input data covering the entire Baltic Sea region. Therefore, the information about specific visible plastics and indicator item emissions of the Warnow Estuary were complemented by literature information and combined with data on wastewater amounts (stormwater, sewer overflow and untreated wastewater) for the entire Baltic Sea catchment. The resulting emissions data on visible plastics and indicator items allowed Baltic-wide model simulations on plastics transport, behavior and deposition. The monitoring at Baltic Sea beaches served for validating the model results and assessing retention of plastics in the estuary.

### Emission Calculations for Rostock city: amounts and Indicator items

**Fig. 3:**
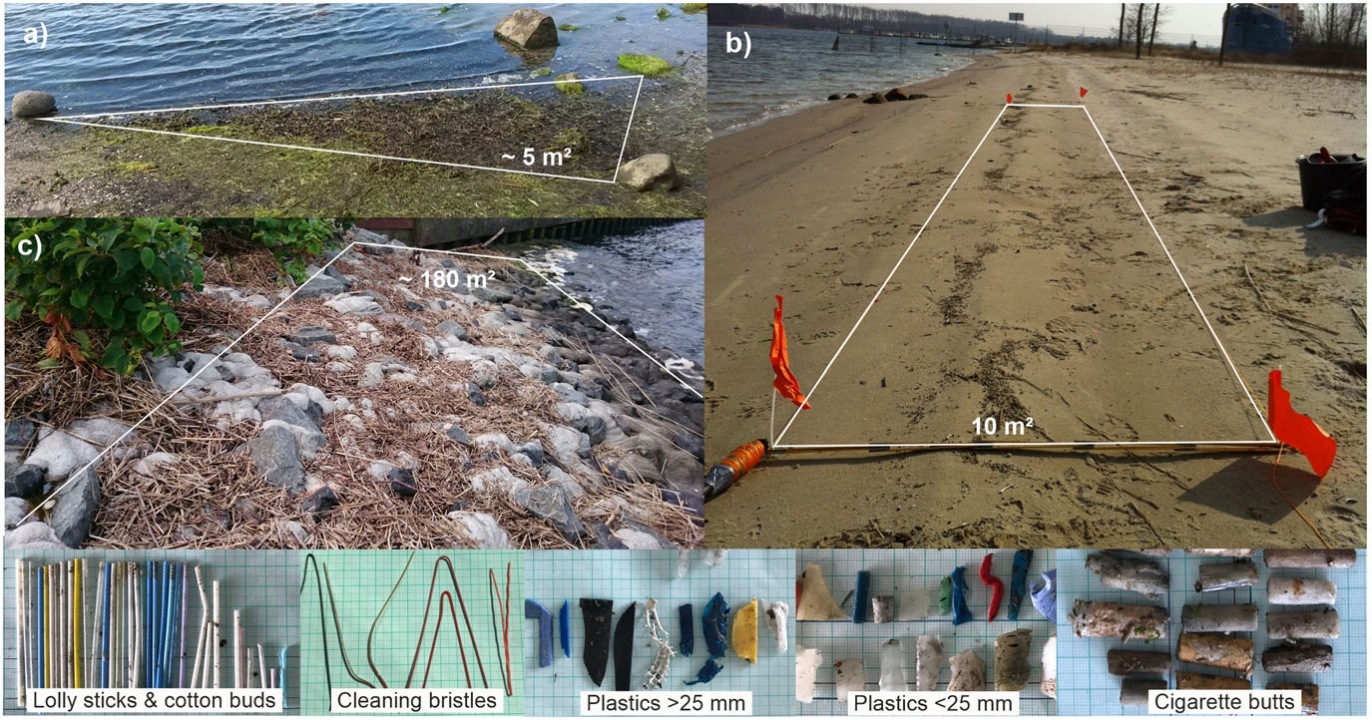
Sampling locations and methods. **a** Schnatermann, **b** IGA-Park, **c** Ottos Steine as well as examples of most commonly found items

Plastic litter in the environment consists of a wide spectrum of size classes, types, shapes and properties. Further, the composition of particles varies between pathways and locations. To reduce uncertainties, to enable reproducible emission calculations, and to allow concrete model simulations on transport and behavior in the sea, we focused on defined plastic litter items. Criteria for the choice were supra-regional and policy relevance, quantity in the environment, representation in monitoring programs and databases, the allocation to and representation of defined pathways and diverse material properties. Cigarette butts (cellulose acetate, CA, 1.3 g/cm^3^), lolly sticks, cotton buds (polyethylene, PE/polypropylene, PP; 0.89–0.97 g/cm³ density) and bristles from street cleaning equipment (polyvinylchloride, PVC; 1.3–1.45 g/cm³ density) turned out to be suitable (Fig. 3).

Rostock city center is connected to a combined sewer system (storm- and wastewater combined), while most of the 181.4 km^2^ city catchment area are connected to a separate sewer system (storm- and wastewater separated). Rostock’s wastewater is treated in the central wastewater treatment plant (WWTP), which includes a mechanical and an activated sludge treatment, followed by a two-step nitrification and denitrification. In the estuary, a total of 167 wastewater outlets were identified including the WWTP outlet, 11 combined sewer outlets and 155 stormwater outlets. As soon as heavy rain exceeds the hydraulic capacity of the combined sewer system, storm- and wastewater are entering the estuary largely without treatment. According to Piehl et al. (2021), the total annual amounts emitted to the estuary were about 41 million m³ stormwater, 107,065 m³ combined sewer overflow water and 16.5 million m³ treated wastewater.

Our emission calculations for large micro-, meso- and macroplastics utilized the data on pathways in Piehl et al. (2021). In the sewer system of Rostock, no traps or sinks for micro- or meso-litter are implemented. Emissions via combined sewer overflows considered the toilet flushing behavior of litter items in general and item specific emissions (Elliott and Elliott 2018). In addition, diffuse and direct emissions by human activities to the estuary were taken into account. Major sources of information were literature, official statistical data and local expert knowledge. First assumptions and calculations were reassessed by an expert group. This group consisted of 11 experts with local knowledge, different backgrounds (biology, physics, economics, ecology, coastal management), different experience and knowledge levels (master degree, PhD, professorship). The expert assessments made use of a Mentimeter polling (https://www.mentimeter.com). Mentimeter is an online interactive presentation software and allows for anonymous and real time voting as well as commenting (Little 2016). In a first step, data, assumptions and calculations were presented online. Afterwards the experts could provide their view online, and assess whether, and if so, to which degree, they considered the emission as under- or overestimated. If justified, the assumptions emission calculations were modified accordingly.

Cigarette butts: for calculating the total emission into the environment per year for the sealed city area of Rostock, we assumed that items emitted within one km buffer around the estuary shoreline (21.77 km² area) eventually end up in the estuary via wind or storm water transport. We took into account the population of the city above an age of 15 years of 180,000 (HRO Statistics 2019), the smoking rate of the German population of 23.4% and an average daily consumption of cigarettes by smokers of 9.7 (Seitz et al. 2019). We assumed that 50% of the cigarettes were smoked outside and that 56% of these were littered to the environment (Miller and Burbach 2017; Rath et al. 2012; Green et al. 2014).

In a second step, depending on the entrance point, we separated between emissions via combined sewer overflows, via storm water runoff, and directly into the estuary. Emissions via storm water runoff assumed a reduction factor due to street cleanings, traps in gullies etc. and considered only butts emitted within a 100 m zone to the shore. The calculations resulted in the following annual numbers: about 150 million cigarettes are smoked in Rostock; 41 million cigarette butts are emitted to the environment in the whole city area (28%) and about 3.6% of the total number are emitted within 1 km to the coastline.

Lolly sticks: similar to cigarette butts, emissions via combined sewer overflows, storm water runoff and directly into the estuary were taken into account and the calculations followed the same principle. It was assumed that the young population (60,000 inhabitants) consumed 18 lollies per year which resulted in a total annual consumption in Rostock of about 1 million sticks.

Cotton buds are mainly used for ear cleaning and makeup. Elliott and Elliott (2018) estimated an annual consumption of 150 cotton buds per capita, which equals a consumption of 27 million buds per year in Rostock. We assumed that they enter the estuary exclusively via combined sewer overflow. We assumed a 4% share of the population that flushes cotton sticks the toilet and the quantitative role of overflow.

Cleaning bristles: Only wash off from sealed city areas (within 1 km from the shoreline) and emissions via stormwater inlets were considered. We assumed that 12 cleaning bristles per square kilometer were emitted by cleaning operations, taking place on 261 days per year and that 30% of the bristles from street cleaning equipment were emitted to the estuary.

### Warnow River and Estuary Surface Monitoring

To quantify the riverine emissions, we carried out a monitoring at the river mouth. On 13 dates between July and September 2017, a monitoring of large micro and mesolitter took place at a total of 9 locations along the Warnow river. In Rostock, at the entrance of the river into the estuary (Fig. 1), samplings took place on 10 days. Depending on the location, we used different net types, a conical 200 µm plankton net, as well as 300 µm and 1000 µm rectangular drift nets, which varied in opening size. The nets were employed to collect plastics drifting on the surface and in the upper water body. Three test samplings with nets fixed on the river bottom were carried out to sample plastics transported on the sediment surface. The exposition time varied between 15 min and 12 h, depending on the mesh size and the amount of organic material in the river. The water flow near the river mouth varied between 0.05 and 0.46 m³/s and, in total, about 2800 m³ of river water were filtered by the nets during the samplings. Water discharge was measured with a CTD and is based on gauge data of StALU MM/WM. The surface water of the estuary was sampled once close to the city center, close to the river mouth, using the same equipment. The net was attached to a crane and dragged beside the boat over 12 min at a speed of 3.8 knots. Nearly 100 m³ water were filtered.

Since focus was on particles above 1 mm, the samples were filtered through a cascade of 1000 and 500 µm metal sieves. The 500 µm limit marks the size of particles still adequately visually and mechanically detectable. Larger fractions of organic matter were rinsed with clean water to flush off attached plastic particles and sorted out. Afterwards, the sieves were visually analyzed, first with naked eye and then under binocular microscope. Potential plastic particles were sorted out using a metal tweezer and placed on a glass Petri dish or slides. The type of plastic material was analyzed with the mobile near infrared spectrometer (microPHAZIR RX). Recovery experiments with 500 µm and 1000 µm sized particles of different colors were carried out to ensure reliable results. The average recovery rate was 90%. Samples analyzed for microplastics below 500 µm were treated with 30% H_2_O_2_ and incubated about 50 h at 50 °C to remove organic material.

### Warnow Estuary Sediment Screening

Besides the coastline, the estuary sediments can potentially be a sink and accumulation areas for plastics. This is especially true for the shipping channels which act as trap for muddy organic rich material. In 2018, harbor authorities removed about 44,661 m³ sediments from the channel in the southern, city part of the estuary and dumped it on a nearby polder. The muddy sediment contained about 75% water and our monitoring took place in September 2019 on the dumping site when most water had been drained off. The sediment surface area was separated into transects and about 3.8% of the total area was screened visually with bare eye method (Haseler et al. 2019) for plastic pieces. The pieces were classified using the OSPAR item protocol and analyzed with the mobile near infrared spectrometer (microPHAZIR RX). For the allocation of the plastic sources, the Matrix Scoring Technique (Tudor & Williams 2004; Haseler et al. 2019) was used.

### Flood Accumulation zone and Estuary Coastline Monitoring

The Warnow estuary and Baltic coastline monitoring formed the core of our data collection activities. Between 2014 and 2017, altogether 34 samplings at five Baltic Sea beaches took place. In addition, ten samplings without sieving were carried out at beaches, especially after storm and high-water level events. For sampling visible plastics and indicator items in flood accumulation zones of beaches, a 10 m long and 1 m wide transect of the tidal accumulation zone was spatially defined. Beach wrack (or tidewrack) within this area was collected first, washed with water and analyzed for plastic pieces by bare eye. Afterwards, a 3 cm sand surface layer was removed and washed through a 2 mm mesh-size metal sieve. The particles remaining in the sieve were identified and collected by bare eye. If possible, the collected particles were analyzed with a mobile near infrared spectrometer (microPHAZIR RX) afterwards. For details about the method see Haseler et al. (2019).

Between 2015 and 2017, this flood accumulation zone monitoring method was applied to the five existing beaches in the estuary, as well. Altogether 10 samplings took place at Schnatermann (54.173 N, 12.142 E), IGA Park (54.139 N, 12.087 E), Oldendorfer Tannen/Gehlsdorf Fähre II (54.100 N, 12.114 E), Hundsburg (54.131 N, 12.090 E) and Ostbreitling (54.163 N, 12.137 E).

To get a more complete picture of plastics accumulated at the coast, four other accumulation areas including stony hard structures were sampled by bare eye for visible plastics: Ottos Steine (54.094 N, 12.113 E), Warnow Hotel (54.096 N, 12.152 E), Gehlsdorf Fähre I (54.099 N, 12.114 E), Holzhalbinsel (54.094 N, 12.150 E). Altogether 12 samplings were carried out, mostly one or a few days after heavy rain events with sewer overflows. Since the estuary monitoring included the flood accumulation zone monitoring and additional simplified approaches, we call it estuary coastline monitoring.

Based on the estuary coastline monitoring, the total annual shoreline pollution in the estuary with large micro- and mesoplastics as well as special items was estimated. In a first step, average concentrations per meter coastline for items and plastic size classes were calculated. Beside hard structures (e.g., quays and walls) that do not allow accumulations, the estuary shoreline is covered by 15 km reed belts, 4 km of stony areas, and 2.4 km beaches. The average plastics concentration for each shoreline type was multiplied with its shoreline length. For reed belts, we assumed the same accumulation behavior as for beaches.

In Rostock in 2016/2017, local heavy rain events with sewer overflow took place on 72 days per year (data provided by EURAWASSER, pers. com.) and caused raw sewage water emissions to the estuary (at least) at one out of the 11 combined sewer outlets. We assumed that the accumulation at the coast takes place only during heavy wind events with increased water levels and that re-suspended plastic material and deposits are found in defined high water accumulation zones. According to DWD weather and water level statistics, in 2017 events with lasting wind above 10 m/s took place 10 times per year.

### Baltic Wide Emissions Scenarios: quantity, location, and items

Model simulations about the transport, behavior and deposition of plastics in the aquatic environment require the concentrations of plastics in and the amount of discharge water as model input. Combined sewer systems, which are common in urban areas of the Baltic Sea region, collect surface water runoff, domestic sewage and industrial wastewater. Baresel and Olshammar (2019) compiled data about and quantified the amount of sewage water for 3525 WWTPs in the Baltic Sea region. Different to model simulations in Schernewski et al. (2020, 2021) we assumed that large microplastics and mesoplastics are fully kept back in WWTPs. As a consequence, treated wastewater was not considered as a pollution pathway.

In combined sewer systems, sanitary sewer overflows take place, where untreated wastewater together with stormwater is discharged into the aquatic environment. Usually this happens due to a temporary insufficient hydraulic capacity after heavy precipitation. Baresel and Olshammar (2019) assumed that in the Baltic Sea region, weather related sanitary sewer overflow without treatment in a WTTPs, accounts for 1.5% of the total WWTP inflow. This value was applied and for seasonal overflow calculations, we assumed that this is equivalent to 1.5% of the time of the year. We assumed that sanitary sewer overflows resulting from technical problems are included in the 1.5%. Separated sewer systems collect storm water and wastewater in separated systems. In these systems, stormwater is always released directly to the aquatic environment usually without treatment. For the Baltic Sea region, reliable numbers about the water discharge from separated sewer systems are lacking. We assumed that separated sewer systems have a share of 50% in the Baltic Sea region. In our model simulations we combined separated and combined sewer systems. Therefore, only two pathways are considered: stormwater together with sewer overflow and untreated wastewater.

The density of plastics (artificial polymers) is an important parameter that determines its transport, behavior and deposition in the aquatic environment. According to the density, we separate floating and sinking polymer types. The group of floating polymers includes low and high density polyethylene (PE, 0.915–0.97 g/cm³ density) and polypropylene (PP, 0.89–0.92 g/cm³ density). The group of sinking polymers covers rigid polyvinyl chloride (PVC, 1.3–1.45 g/cm³ density), cellulose acetate (CA, 1.3 g/cm^3^), and polyethylene terephthalate (PET, 1.38 g/cm³ density). The most common polyester fibers are made of PET. Acrylic and polyamide fibers have a density of only 1.18 g/cm³ resp. 1.14 g/cm³. According to Sun et al. (2019), PP, PE, and PET are the most abundant polymers in WWTPs.

Previous model simulation studies (Schernewski et al. 2020, 2021) did show that the separation into two density classes is sufficient to reflect the behavior of the vast majority of plastics in the marine environment. Beside density, floating and sinking behavior of microplastics is influenced by particle size, shape and processing. We did not take into account particle size and shape, but with respect to cigarette butts the processing had to be considered.

Because of the high air content, cigarette filters, usually made of cellulose acetate fiber, are floating. To assess cigarette butt behavior in water, we carried out simple experiments. We put cigarette butts, consisting of the filter, paper (cellulose) and residual tobacco in glass cylinders filled with Baltic Sea water and exposed them for one week to sunlight under daily stirring. After six days, only four out of 17 cigarette butts were still floating, while the rest fragmented to its components and sunk to the bottom. The sinking of the soaked pure cigarette filters was tested in large glass cylinders and resulted in relatively high sinking velocities between 0.03 and 0.07 m/s.

### Model Approach and Simulations

The modeling approach followed Osinski and Radtke (2020a). We used the UERRA high-resolution atmospheric reconstruction, provided by SMHI, to drive both a third-generation wave model (WAVEWATCH 3) and a hydrodynamic model for the Baltic Sea (GETM). Both models have a horizontal resolution of one nautical mile. A microplastics transport module was added following Osinski et al. (2020b). The hydrodynamic model provided the current field used for the passive transport of the particles, which are represented in a Eulerian framework as a concentration per grid cell. The size, density and shape of the particles determined the vertical velocity relative to the ambient water and the critical shear stress for the resuspension. The actual shear stress at each time step was calculated from the bottom current velocity and the significant wave height which was provided from the wave model. Settled particles were re-suspended when the actual shear stress exceeds the critical value.

The shape is a factor that determines the behavior of particles in the aquatic environment (e.g., Kooi and Koelmans 2019; Kowalski et al. 2016). However, we did not distinguish between fragments, beads, spheres, flakes and films but partly separate fiber. Our previous study (Schernewski et al. 2020) did show that differences in shape and size only have a limited effect on our simulation results. This is only true for our the chosen temporal and spatial resolution of our model approach. Sinking velocities were determined from the Stokes parameterization assuming a spherical shape. For each size class, we used the lower size limit as the particle diameter assumed for the Stokes formula, since (a) smaller particles typically have a higher abundance and (b) deviations of larger particles from the spherical shape would cause a reduction in the vertical velocity, such that they would behave like smaller spherical ones in this sense. The critical shear stress was calculated from the Shields curve (Shields 1936). For fibers we took the empirically determined parameterizations by Waldschläger and Schüttrumpf (2019a) for the sinking velocity and Waldschläger and Schüttrumpf (2019b) for the critical shear stress. The vertical model resolution and the calculation time steps did not allow the correct representation of heavy, fast sinking particles. Because of our choice of particle size classes and items, this problem was negligible.

Particles entering a grid cell adjacent to a land cell were immediately removed from the model and counted as beached. The beached particles were accumulated over a year to provide numbers of the total amount of particles washed ashore. An exception were those grid cells acting as a source, such as rivers, here we did not assume a beach accumulation. A possible resuspension and further transport of the particles was neglected. The model simulations altogether covered two years. The period from March 2016 until February 2017 was used for calculating annual values.

Our model approach allows a scaling of the MP concentrations in the environment, by post processing the simulation results. This means the absolute concentrations emitted via each pathway and size class potentially can be adjusted if new insights or better field data are available. This is possible as long as the relative spatio-temporal emission pattern remains the same.

## Results

### Rostock city Plastic item Emissions to the Warnow Estuary

Emissions of microplastic with waste- and stormwater from the city are addressed and documented in Piehl et al. (2021). The emission of visible plastics via these pathways can hardly be estimated based on literature and calculations, because visible plastics can have multiple sources and can stem from fragmentation. Here we focus on emission estimations for the indicator items cigarette butts, lolly sticks, cotton buds, and cleaning bristles. The annual cigarette bud emission rates to the estuary were: 534,000 direct emissions, 961,000 via stormwater runoff and 368,000 via combined sewer overflows. The resulting estimates for Rostock were that about 1,860,000 cigarette butts end-up in the estuary annually or 1.25% of the annually smoked cigarettes.

About 3900 lolly sticks were calculated to be emitted directly to the estuary, 7000 enter via stormwater and 1100 via combined sewer overflow. In total about 12,000 sticks or 1% of the consumed lolly sticks were assumed to enter the estuary. With respect to cotton sticks we received an emission to the estuary of about 11,000 or 0.04% of the annually consumed cotton buds in Rostock. With respect to cleaning bristles we calculated a total emission to the estuary of about 20,000 bristles per year.

### Warnow River Plastic Emissions to the Estuary

The Warnow river is the only major river discharging to the Warnow Estuary. All minor riverine systems are part of the Rostock city drainage system. Piehl et al. (2021) analyzed microplastics emissions to the Warnow Estuary (based on very few data) and assumed that more than 1/3 of the total emissions enter with the river. This means that the river potentially can be an important source for large micro- and mesoplastics, as well. In the Warnow river, altogether 36 phytoplankton-net samplings using mesh-sizes of 200, 300, and 1000 µm were carried out. Above 100 m³ estuary water were filtered using the 300 µm drift net. The discharge of the Warnow river at the mouth was about 15 m³/s and in 2017 in average and varied seasonally between 7 and 25 m³/s.

In about 70 m³ river water passing the 200 µm net, 23 potential plastic particles were found, resulting in a concentration of 0.32 particles/m^3^. Our methodology did not allow to verify that these were plastic particles because they were too small for the analysis with the mobile infrared-spectrometer. Large amounts of organic material and the clogging of the nets allowed only exposure times below 1 h and caused problems in sample treatment. With the 300 µm and 1000 µm nets about 1000 m³ river water were filtered with each net. The obtained numbers of particles were 18 (300 µm net) and 7 (1000 µm net). The resulting concentrations of potential plastic particles at the river mouth were 0.016 resp. 0.006 particles/m³. Only less than 50% of the particles could, without doubt, be identified as plastics using the mobile infrared spectrometer. The dominating plastic type was polyethylene followed by polypropylene. About 70% of all particles were smaller than 1 mm, 25% between 1 and 5 mm, and 5% larger than 5 mm. The data obtained from samplings at locations upstream was quantitatively not sufficient for an analysis.

Based on the results from the 300 µm net, the calculated annual plastics discharge of the Warnow river were about 3 million plastic particles. Including the unidentified, potential plastic particles, the annual discharge was 7.7 million plastic particles. For the size class between 1 and 25 mm, the annual load was 3 million potential plastic particles, including about 1 million identified PE and PP particles. This weak data basis and the fact that none of the indicator items (cigarette butts, lolly sticks, cotton buds, and cleaning bristles) were found, did not allow for reliable and transferable assumptions on the role of the river as pathways for large micro- and mesoplastics and the selected items.

### Plastics in the Warnow Estuary: water body

We carried out a monitoring in the estuary, covering the water body and the coastline. One major aim was to assess whether the monitoring results match to and verify our emission calculations for the indicator items. Further, we wanted to get an impression of type, size, form and quantity of other visible plastics items in the estuary.

The water surface was sampled once with a 300 µm net dragged aside a boat. A total of 15 potential plastic particles were found in nearly 100 m³ water. Six particles could be reliably identified as plastics, mainly polyethylene and polystyrene. The concentration in estuary water was 0.14 potential plastics particle per m³ and 0.05 identified plastic particles per m³. Indicator items were not found. The relatively low number of particles found, the restriction to floating plastics and the relatively high effort point out the weaknesses of this method for our purpose. As a consequence, the method was not further applied.

### Plastics in the Warnow Estuary: Sediments

Sediments can serve as a sink for plastics and potentially can provide some insight with respect to plastic types and pathways. Altogether, 1371 plastic pieces were found on 1723 m² on the dumping site for dredged muddy channel sediments. The most abundant were plastic fragments (32.6%), followed by flat pieces of plastic (strapping bands, 20%), cups and cup lids (6.3%), corrugated plastic (5.9%), food containers incl. fast food containers (5.6%), and sanitary towels/panty liners/backing strips (4.7%). The main polymer types of the identifiable 804 items, were polyvinyl chloride (24.4%); polypropylene (15%), polyethylene (10%); polystyrene (7.8%), and polyamide (6.1%). With the expert-based Tudor Matrix Scoring System (Tudor & Williams 2004), residents and tourists were assigned to be the source of 26% of the litter, followed by Combined Sewer Overflow emerges with (19%), shipping (15%), angling and fishing (15%), construction (14%), and industry (11%).

By far most particles found in the sediments belonged to the macroplastic fraction (>25 mm) and the method did not allow for a quantification of the sediments as sink for plastics below 25 mm. Despite that, several relevant conclusions can be drawn: Stormwater and sewer overflow is one of the most important plastic pathway, the organic channel sediments serve as important sinks for plastics and not only for sinking items with a density above 1 g/cm². The high share of PP and PE particles indicates that biofilms, microorganisms, algae and detritus settled on plastics, increase the specific weight and enable an accumulation of originally buoyant plastics in the sediment. The muddy character of the sediments indicates that deeper channel are protected from wave induced resuspension. Otherwise these sediments including plastics would have been accumulated at the shore or washed into the Baltic Sea.

### Plastics in the Warnow Estuary: Coastline

Results in Schernewski et al. (2020, 2021) indicate that plastic particles with a density between 0.8 and 1.4 g/cm³, which constitute the vast majority of all plastics in the environment, are washed ashore within days after emission to the Baltic Sea. Consequently, our monitoring focused more on the coastline, instead of water column and sediments. This ensured that a wide range of plastic types and size classes could be monitored at many locations in a cost- and time-efficient way. However, the coastline structure did not allow the application of one consistent method.

Table 1 provides a full overview about the coastline monitoring. Altogether over 2300 items were found. With 411 pieces (18% resp. 1.7 items/m²) cigarette butts were most abundant, followed by 278 plastic pieces (unidentifiable fragments) between 5 and 25 mm (12% resp. 1.0 item/m^2^) and 231 larger plastic pieces between 25 and 500 mm. The average number of particles per m² was 0.7. The indicator items, cleaning bristles (6%) cotton buds (3%), and lolly sticks (0.7%), had much lower shares. 133 cleaning bristles were found at Schnatermann during one sampling, but rarely at other locations. Therefore, this item was not further considered as suitable indicator item for pollution.

**Table 1:** Partly aggregated Warnow Estuary coastline monitoring data

Sampling spot	Southern stones	Southern beaches	Ottos Steine	Schnater-mann	Schnater-mann	Schnater-mann	IGA Park	IGA Park	Alter Strom	Marina		
Sampling method	bare eye	bare eye	bare eye	bare eye	bare eye	sieve	sieve	bare eye	bare eye	bare eye		
Number of samplings	3	3	4	1	2	3	1	1	1	1		
Water level (above MWL)	96 cm	96 cm	0–30 cm	15 cm	96 cm	15–94 cm	7–95 cm	153 cm	153 cm	153 cm	Average	

OSPAR item name	% (items/m^2^)										%	/m^2^
Cigarette butts and filters	16.3 (0.47)	37 (2.83)	12 (0.22)	7.3 (4.6)	6.4 (1.1)	4 (0.43)	27.1 (1.17)	6.6 (0.6)	14.1 (5.1)	1.2 (0.3)	17.8	1.68
Plastic pieces 5–25 mm	11.6 (0.33)	3.5 (0.27)	7.7 (0.14)	0 (0)	2.9 (0.5)	23.4 (2.53)	8.5 (0.37)	38.5 (3.5)	5.5 (2)	2 (0.5)	12.1	1.01
Plastic pieces 25–500 mm	0 (0)	2.2 (0.17)	5.7 (0.11)	14 (8.8)	1.7 (0.3)	13.2 (1.43)	6.2 (0.27)	13.2 (1.2)	8.9 (3.2)	2 (0.5)	10.0	1.60
Fireworks plastic pieces	2.3 (0.07)	0.9 (0.07)	3.5 (0.06)	11.1 (7)	0.6 (0.1)	0 (0)	0.8 (0.03)	2.2 (0.2)	26.9 (9.7)	1.2 (0.3)	8.2	1.95
Polystyrene pieces 25–500 mm	5.8 (0.17)	6.1 (0.47)	0.8 (0.01)	0 (0)	24 (4.15)	0 (0)	0 (0)	0 (0)	1.7 (0.6)	49.6 (12.5)	10.5	2.56
Plastic pieces <5 mm	0 (0)	0 (0)	0 (0)	0 (0)	0 (0)	12.9 (1.4)	19.4 (0.83)	0 (0)	0 (0)	0 (0)	2.9	0.32
Industrial pellets	0 (0)	5.2 (0.4)	0.6 (0.01)	0 (0)	0 (0)	7.1 (0.77)	5.4 (0.23)	0 (0)	0 (0)	0 (0)	2.2	0.20
Crisp packets/sweet wrappers	9.3 (0.27)	5.7 (0.43)	5.7 (0.11)	0.3 (0.2)	5.5 (0.95)	2.2 (0.23)	0.8 (0.03)	6.6 (0.6)	4.7 (1.7)	5.2 (1.3)	7.0	0.58
Bags incl. Pieces (all)	2.3 (0.07)	0.4 (0.03)	0.4 (0.01)	0 (0)	0 (1)	8 (0.87)	3.1 (0.13)	0 (0)	5 (1.8)	2 (0.5)	2.6	0.44
Foam sponge	0 (0)	0.4 (0.03)	0.7 (0.01)	0 (0)	0 (0)	1.8 (0.2)	0.8 (0.03)	3.3 (0.3)	0 (0)	13.9 (3.5)	2.4	0.45
Plastic caps and lids (all)	0 (0)	0.9 (0.07)	3 (0.06)	0 (0)	1.4 (0.25)	0.6 (0.07)	0 (0)	0 (0)	5.3 (1.9)	4 (1)	3.4	0.33
Foil wrappers, aluminum foil	0 (0)	1.7 (0.13)	1 (0.02)	2.2 (1.4)	0.3 (0.05)	1.5 (0.17)	3.9 (0.17)	5.5 (0.5)	0.6 (0.2)	2 (0.5)	2.0	0.31
String & cord (diam. <10 mm)	1.2 (0.03)	1.7 (0.13)	1.2 (0.02)	0.3 (0.2)	0 (0)	1.8 (0.2)	2.3 (0.1)	1.1 (0.1)	0.6 (0.2)	0 (0)	1.5	0.11
Food contaimers e.g., fast food	4.7 (0.13)	0 (0)	2.8 (0.05)	0 (0)	0 (0)	0 (0)	0.8 (0.03)	3.3 (0.3)	0.3 (0.1)	7.9 (2)	2.9	0.29
Bottle caps, lids, & pull tabs	0 (0)	0 (0)	0.9 (0.02)	0.3 (0.2)	0 (0)	0.9 (0.1)	0.8 (0.03)	2.2 (0.2)	0.8 (0.3)	1.6 (0.4)	1.1	0.13
Lolly sticks	1.2 (0.03)	0.4 (0.03)	0.3 (0.01)	0 (0)	0 (0)	0.3 (0.03)	0 (0)	6.6 (0.6)	0.8 (0.3)	0 (0)	0.7	0.10
Cups and cup lids	2.3 (0.07)	0 (0)	0.2 (0)	3.2 (2)	0 (0)	0 (0)	0 (0)	0 (0)	1.4 (0.5)	0 (0)	0.9	0.26
Cleaning bristles	0 (0)	0 (0)	0.1 (0)	42.2 (26.6)	0.3 (0.05)	0 (0)	0 (0)	0 (0)	0 (0)	0 (0)	5.9	2.67
Cotton buds	4.7 (0.13)	1.3 (0.1)	4.4 (0.08)	0 (0)	0 (0)	0 (0)	0 (0)	0 (0)	0 (0)	0 (0)	2.9	0.03
Other sanitary items	2.3 (0.07)	0.4 (0.03)	3.3 (0.06)	1 (0.6)	0 (0)	0 (0)	0 (0)	0 (0)	0 (0)	0 (0)	2.2	0.08
Medical Items	0 (0)	0 (0)	1.1 (0.02)	0.3 (0.2)	1.7 (0.3)	0 (0)	0 (0)	0 (0)	0 (0)	0 (0)	1.0	0.05

“Southern stones” include Gehlsdorf Fähre, Warnow Hotel and Holzhalbinsel. Southern beaches cover the sampling spots Oldendorfer Tannen and Gehlsdorf Fähre

### Total Plastics Emissions to the Warnow Estuary

Based on the data in Table 1 and the information on coastline length and structure in the estuary, the total annual accumulation at the coastline was estimated to allow a comparison with emission calculations. For cigarette butts we received a total annual coastal accumulation of 1.35 million compared to 1.9 million estimated emitted cigarette butts. The calculated annual coastal accumulation for cotton buds is 79,000 and for lolly sticks 82,000. This is about seven times higher compared to the estimated annual emitted numbers (12,000 resp. 11,000).

We can summarize that items such as cigarette butts, cotton buds and lolly sticks may serve as indicators for sewage and stormwater related plastic inputs, the emissions can be estimated and a coastline monitoring allows to estimate the annual coastal accumulation within the estuary. However, the emission calculations are based on several assumptions and have a high uncertainty. The coastal monitoring methodology has several weaknesses and inconsistencies (e.g., application of different methods) and the data shows a strong spatial and temporal variability. The extrapolation to annual data for the entire estuary adds several uncertainties. Despite that, the comparison of the annual emission and accumulation data for each item is in the same order of magnitude. The data is hardly reliable, but it seems that we met the dimension of the pollution with these items.

The general agreement between emission estimates and coastal accumulation data for the indicator items allows the assumption that the coastline monitoring can give an insight into the emission of other items and plastic size classes, specified in Table 1, as well. The annual emissions to the estuary for plastic size classes and other items cannot be estimated directly, similar to the approach used for the indicator items. This would mean, for example, that the calculated annual number of visible plastic particles between 5 and 25 mm accumulated at the coastline of about 2.5 million pieces meets the dimension of the annual emissions. Together with the plastic fraction below 5 mm, we would get an annual emission above 3 million plastic pieces.

The catchment of the Warnow river has a similar population as Rostock city and the sewage and stormwater management is comparable. As a consequence, the emission of plastics via the river should be similar compared to the emissions from Rostock city, as long as no particles are retained in the river. No indicator items were found during river sampling, but it remains unclear if this results from retention in the river or from the weak data basis and low number of particles found. Assuming that the few data for visible plastics at the river mouth allows an extrapolation, the plastic input with the river of about 3 million particles/a would be comparable to annual amount accumulated at the estuary coastline of 2.5 million pieces. On the other hand, the plastic concentrations at the river mouth were with 0.016 particles/m³ compared to 0.14 particles/m³ in the estuary much lower. Our weak data basis cannot settle the question whether and to what degree retention of visible plastic particles takes place in the river.

The data on visible plastics and indicator items in the river, emission from the city and accumulation at the coastline was compiled and spatially up-scaled for the model simulations. Because of the uncertainties with respect to plastic retention in the Warnow river, the model approach neglected retention. This approach is supported by Labrenz (2020) who reports an increasing microplastic concentration from the upper Warnow river towards the mouth.

### Plastic Accumulation at Baltic Sea Coasts

While the data from the estuary served as model input, the micro- and mesoplastic monitoring at beaches located in the surrounding of the Warnow Estuary but facing the open Baltic Sea served the spatial model validation. Previous model simulations on small microplastics indicated that most coastal accumulation takes place close to the emission spot with a strong decline of concentrations with increasing distance, but the results were not validated by data (Schernewski et al. 2020, 2021). Therefore, the sampling spots were chosen to catch potential accumulation hot-spots as well as the spatial gradients along the coast. Driving questions were: is the estuary a major source for large micro- and mesoplastics, if yes, is it true that it is washed ashore shortly after the emission and close to the plastics emission points and do strong spatial gradients exist?

The Baltic Sea beach monitoring data (Table 2) shows the highest density of particles per sampling for Warnemünde beach (7–31 particles/m²), followed by Hohe Düne (6–17 particles/m²), both located close to the estuary mouth. For all other, more remote locations the numbers are much lower Markgrafenheide (1.2 particles/m²) and Kägsdorf, Nienhagen and Darss (0.3 particles/m²). These numbers take into account all visible items in Table 2. The concentrations at beaches within the estuary, that were sampled with similar methods, was 3–8 particles/m², so lower than Warnemünde and Hohe Düne. Indicator items, such as lolly sticks, were only found in Warnemünde and Hohe Düne and at comparable concentration like within the estuary. Similar to the locations within the estuary, cigarette butts and plastic pieces (5–25 mm) were the quantitatively most important item groups. The concentrations of cigarette butts within the estuary and at Baltic Sea beaches near the mouth were highly variable, but above 20 butts/m² at several locations. In average, the share and quantity of plastic pieces (5–25 mm) was higher at Baltic Sea beaches.

**Table 2:** Partly aggregated Baltic Sea beach monitoring data. The locations are indicated in Fig. 4

Sampling spot	Nien-hagen	Kägsdorf	Warne-münde	Warne-münde	Hohe Düne	Hohe Düne	Markgra-fenheide	Darss
Sampling method	sieve	sieve	sieve	bare eye	sieve	bare eye	sieve	sieve
Number of samplings	1	4	7	4	7	3	1	3
Water level (above MWL)	26 cm	16– 41 cm	15–95 cm	153 cm	15–95 cm	153 cm	26 cm	14–24 cm

OSPAR item names	% (items/m^2^)							
Cigarette butts and filters	66.7 (0.2)	11.6 (0.1)	56.8 (5.2)	37.6 (17.4)	17.3 (1.5)	14 (2.5)	8.3 (0.1)	22.2 (0.1)
Plastic pieces 5–25 mm	33.3 (0.1)	4.7 (0.05)	5 (0.44)	0.3 (0.15)	17.6 (1.5)	20.8 (3.8)	16.7 (0.2)	0 (0)
Plastic pieces 25–500 mm	0 (0)	0 (0)	1.6 (0.14)	0.7 (0.33)	9.7 (0.84)	30 (5.43)	0 (0)	0 (0)
Fireworks plastic pieces	0 (0)	0 (0)	6.4 (0.59)	21.2 (9.8)	1.9 (0.16)	0.2 (0.03)	0 (0)	0 (0)
Polystyrene pieces 25–500 mm	0 (0)	0 (0)	0.2 (0.01)	0.5 (0.25)	0 (0)	0 (0)	8.3 (0.1)	0 (0)
Plastic pieces <5 mm	0 (0)	2.3 (0.03)	0.2 (0.01)	0.2 (0.08)	7.6 (0.66)	0 (0)	0 (0)	0 (0)
Industrial pellets	0 (0)	2.3 (0.03)	1.7 (0.16)	0 (0)	0.3 (0.03)	23.5 (4.3)	8.3 (0.1)	0 (0)
Crisp packets/sweet wrappers	0 (0)	0 (0)	1.2 (0.11)	1.6 (0.75)	3.3 (0.3)	3.5 (0.6)	8.3 (0.1)	0 (0)
Bags incl. Pieces (all)	0 (0)	2.3 (0.03)	0.5 (0.04)	0.9 (0.43)	3 (0.26)	0.2 (0.03)	8.3 (0.1)	0 (0)
Foam sponge	0 (0)	0 (0)	0.5 (0.04)	0 (0)	0.3 (0.03)	0.7 (0.13)	0 (0)	0 (0)
Plastic caps and lids (all)	0 (0)	2.3 (0.03)	0.5 (0.04)	2.4 (1.13)	3 (0.26)	1.1 (0.2)	0 (0)	0 (0)
Foil wrappers, aluminum foil	0 (0)	0 (0)	0.8 (0.07)	0 (0)	0 (0)	0.7 (0.13)	0 (0)	0 (0)
String & cord (diam. <10 mm)	0 (0)	2.3 (0.03)	0.9 (0.09)	0.1 (0.03)	5.2 (0.45)	0.6 (0.1)	41.7 (0.5)	27.8 (0.17)
Food contaimers e.g., fast food	0 (0)	0 (0)	0 (0)	0.2 (0.08)	0 (0)	0 (0)	0 (0)	0 (0)
Bottle caps, lids & pull tabs	0 (0)	2.3 (0.03)	3.3 (0.3)	0.4 (0.18)	0.6 (0.05)	0.2 (0.03)	0 (0)	0 (0)
Lolly sticks	0 (0)	0 (0)	0.3 (0.03)	0.5 (0.23)	0.3 (0.03)	0 (0)	0 (0)	0 (0)
Cups and cup lids	0 (0)	0 (0)	0 (0)	0.3 (0.15)	0 (0)	0 (0)	0 (0)	0 (0)
Sum visible plastics/m²	0.3	0.3	7.3	30.9	6.1	17.3	1.2	0.3

**Fig. 4:**
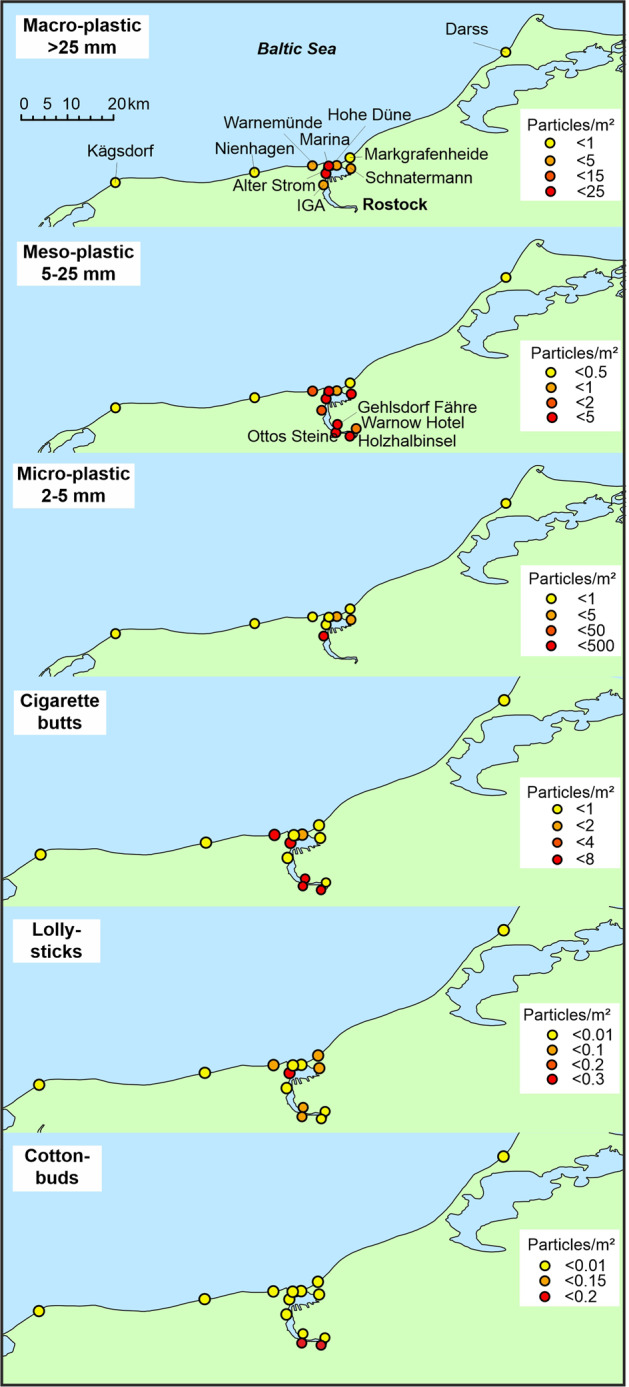
Results of the coastline monitoring for selected items an item groups in the Warnow Estuary and nearby southern Baltic Sea beaches based on data in Tables 1 and 2

The data for selected items are visualized in Fig. 4. Items such as lolly sticks and cigarette butts did show locations with increased concentration in and outside the estuary. Clear gradients within the estuary, between the city center and Baltic Sea, were not obvious. The same was true for mesoplastic (1–25 mm), which shows high concentrations in and around the estuary compared to more remote Baltic Sea beaches. Only cotton buds, the most specific indicator for sewage overflow, was found exclusively near the city center and hardly at other locations.

Altogether we can summarize that the estuary and nearby Baltic Sea beaches are hot-spots for plastic pollution and accumulation. It is likely that urban water-related emission plays an important role, but local pollution from tourism may play an important role, as well. Items such as cotton butts indicate that sewage overflow are relevant pollution events in the Warnow Estuary, too. However, no plastic item or item class is suitable to provide a consistent spatial picture of the transport, behavior and deposition of plastics within the estuary and between estuary and Baltic Sea. Whether the estuary serves as important plastic pollution source for the Baltic Sea cannot settled based on the data. The strong accumulation of plastics at coastlines within the estuary clearly indicates that the estuary coastline serves as a sink for plastics and reduces the load to the Baltic Sea, but this cannot be quantified.

### Emission, behavior, and Coastal Accumulation of visible Plastics in the Baltic Sea

The estuary monitoring data served as model input and the Baltic Sea coast data was meant for model validation. Here, we focus on visible plastics (1–25 mm). Aim of the model simulations was to explain large visible plastic transport, behavior and deposition as well as to address the question of plastic retention in coastal systems. Planned was a two-step modeling approach with a spatially high resolved hydrodynamic model (below 100 m grid cells) in the estuary, linked to a the spatially less refined model covering the entire Baltic Sea. The modeling approach in the estuary failed. As consequence all following results are based on the Baltic Sea model only. The Baltic Sea model with a grid of 1 nautical mile did not sufficiently resolve the estuary.

**Fig. 5:**
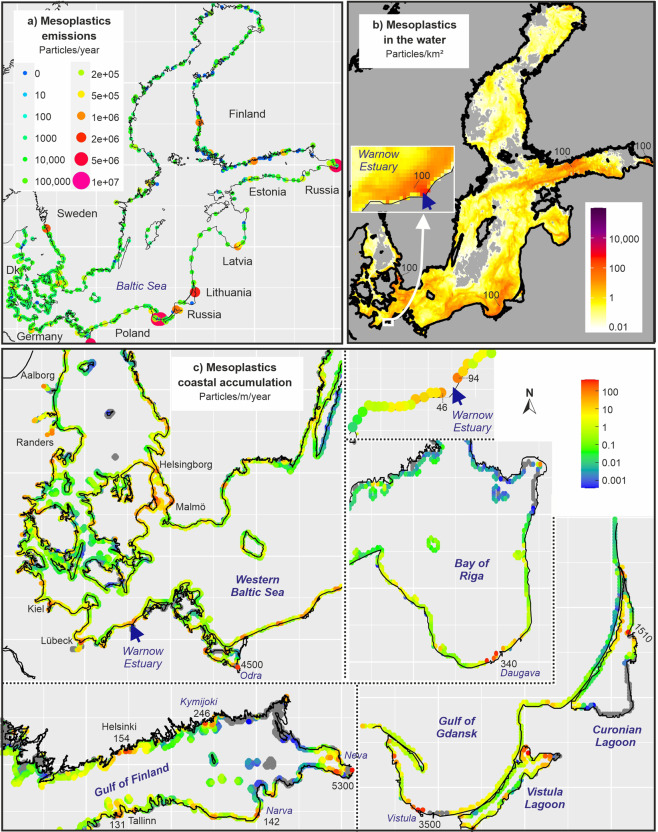
Baltic Sea: **a** emissions of large micro and mesoplastic particles (1–25 mm) from urban sources (untreated wastewater, stormwater and sewer overflow water) to the Baltic Sea assuming no retention in rivers; **b** average annual spatial concentration of plastic particles (1–25 mm size fraction) in the water column and **c** accumulation of plastic particles (1–25 mm size fraction) at different Baltic Sea shores based on simulations with a 3D hydrodynamic model. Gray areas in the sea indicate concentration below the color scale

In most areas of the Baltic Sea, the concentration of visible plastics was below 1 particle per km² sea surface (Fig. 5). Only in the surrounding of major emission spots, usually major river or cities, the model suggested much higher concentrations in larger areas. Examples were areas near Helsinki, St. Petersburg, Gdansk or the Danish Straits were concentrations above 100 visible plastic particles/km² occurred (Fig. 5b). Near the Warnow Estuary, concentrations above 100 particles/km² were restricted to an area of about two kilometers diameter around the mouth. The coastal Baltic Sea is shallow and wave induced turbulence does not allow a permanent settling of plastics with a density above 1 g/cm³ on the predominantly sandy sediments close to the coast. We considered only common plastics with densities between 0.9 and 1.4, which constitutes the vast majority of plastics in the environment. Depending on the plastic density, the residence times of particles in the Baltic Sea differed, but, in average, was below two weeks. A consequence of this short residence time was, that emissions and deposition were balanced already within a few months and stable concentration pattern in the sea were established. Changes in the amount and location of emissions caused modified spatial distribution patterns in the sea, but according to our model, emissions cannot cause an ongoing accumulation of large micro- and mesoplastics in the sea.

The vast majority of emitted plastic particles were washed ashore within days and usually close to the emission spot (Fig. 5c). As a consequence, the model suggested very high annual plastic accumulations near major cities and rivers. Examples were the Odra mouth (Poland) with 4500 visible plastic particles/m coastline per year, St. Petersburg (Russia) with 5300 particles/m/year, the Vistula mouth (Poland) with 3500 particles/m/year or the Nemunas (Lithuania) with 1510 particles/m/year.

For the Warnow Estuary, the model suggested 46 visible plastic particles/m/year (1–25 mm size class in Warnemünde) resp. 94 particles/m/year (Hohe Düne), next to the model emission cell and strongly decreasing concentrations with increasing distance to the estuary mouth. This spatial pattern was consistent with the pattern obtained from monitoring data for this size class (Table 2). Higher accumulations at Hohe Düne compared to Warnemünde were supported by the model and even the absolute numbers were fairly in agreement. This was true, if we assumed that a beach monitoring was carried out every two weeks (the common sampling interval for assessments within the EU Water Framework Directive) and would have always deliver plastic concentrations at the locations as documented in Table 2. However, the direct comparison of monitoring and model data is linked to many uncertainties. The model did not take into account retention in rivers and in the estuary, but to conclude that if model and data match, retention ddid not play a role is certainly misleading. The pollution at the beaches can have local reasons, such as tourism, and was not necessarily resulting from plastics carried by estuarine water.

### Emissions, behavior, and Accumulation of single Plastic items in the Sea

The item group covering large micro and mesoplastic particles (1–25 mm) covered a wide range of plastic types, often fragments, with different properties, shapes and origins. It was not specific enough to provide information about the role of the estuary for the pollution of the nearby Baltic Sea beaches. Cotton buds and lolly sticks have a similar shape, are usually made of PE/PP and are therefore floating. Stormwater and sewer overflow can be regarded as major pathway for these items to the environment. Stormwater and sewer overflow only takes place within the estuary. Therefore, the assumption was that these items can serve as reasonable indicator for beach pollution resulting from the estuary. Because of low numbers, data on cotton buds and lolly sticks of both is aggregated in the following.

**Fig. 6:**
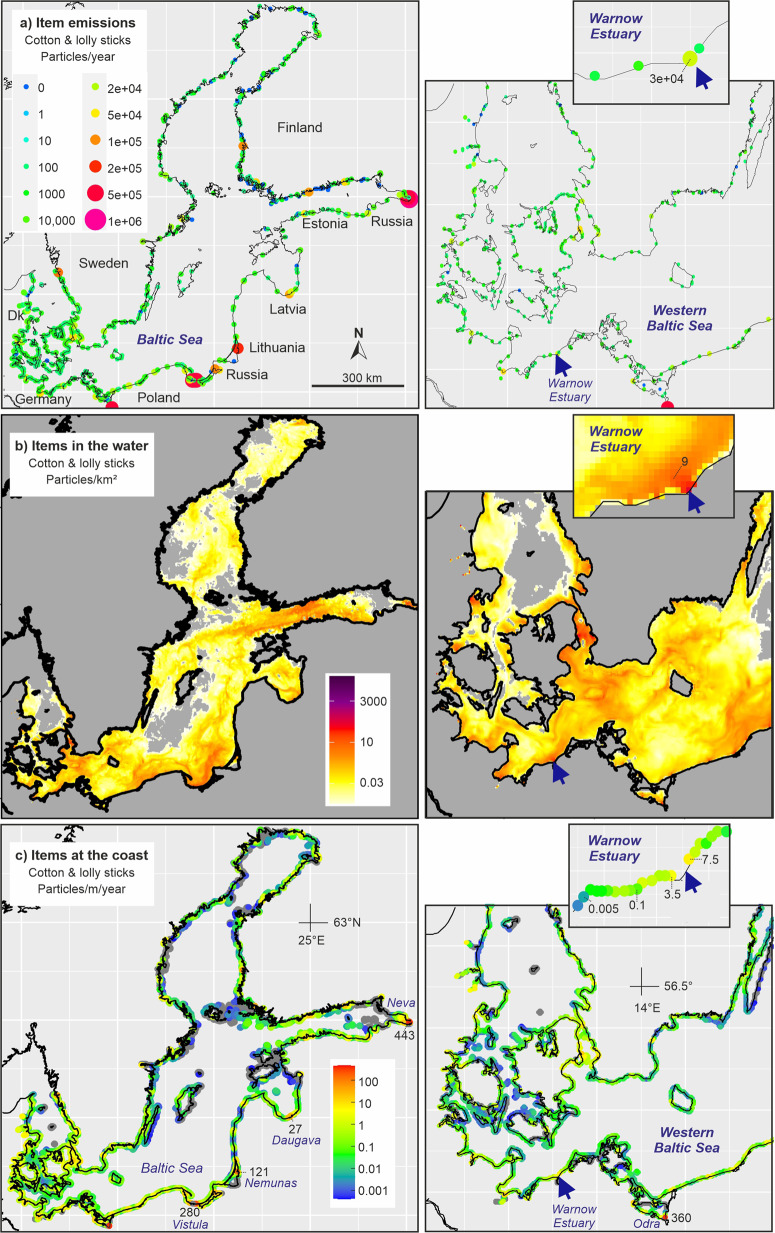
Baltic Sea: **a** emissions of cotton buds and lolly sticks from urban sources (untreated wastewater, stormwater and sewer overflow water) to the Baltic Sea assuming no retention in rivers; **b** average annual spatial concentration of cotton buds and lolly sticks in the water column and (**c**) accumulation of these items at different Baltic Sea shores based on simulations with a 3D hydrodynamic model. Gray areas in the sea indicate concentration below the color scale

Again rivers and large cities were major emission spots and the model suggested concentrations above 1 particle/km² in the sea areas close to the emission spots (Fig. 6a,b). In the Warnow estuary plume, a few kilometers off the coast the model suggested annual average concentrations of 9 sticks/km² (cotton buds and lolly sticks). For coastlines close to the major emission spots, the model suggested annual accumulations that could, in single cases, exceed 100 sticks/m coastline per year. Examples were the Vistula (Poland) with 280 and the Neva/St. Petersburg (Russia) with 443 sticks/m coastline per year. Higher concentrations in eastern Baltic locations resulted from a combination of high population and, compared to northern and western Baltic Sea states, a higher discharge of untreated wastewater.

At Warnemünde and Hohe Düne, beaches next to the Warnow estuary, the model suggested annual accumulations of 3.5–7.5 sticks/m coastline. If we again assume that a beach monitoring is carried out every two weeks and would always deliver plastic concentrations at the locations as documented in Fig. 4, the model results fairly matched the data. However, this statement is based on only few lolly sticks found during the monitoring. Cotton buds were hardly found.

We need to conclude that in areas where larger amounts of untreated sewage water enter the coastal sea, the monitoring of indicator items may be a useful approach to assess the role of urban waste- and stormwater on coastal pollution. For the Warnow Estuary, the number of emitted cotton buds and lolly sticks was too low. The few found sticks at surrounding Baltic Sea beaches can hardly serve as indicator for pollution resulting from urban waste- and stormwater.

### Retention of Plastic items in Rivers and Estuaries

Cigarette butts and filters are, within the estuary and at the Baltic Sea beaches, the quantitatively most important single plastic litter item. We can assume a direct relationship between number of cigarette butts emitted to the environment and the human population (modified by social and behavioral factors). The German Baltic Sea coast is characterized by intensive summer tourism. We can assume that direct local emissions by tourists and local population as well as urban wastewater related emissions were dominating and caused the pollution intensity and pattern observed in the monitoring data. Since our model approach addressed only one of these pathways, we cannot expect a close relationship between model results and monitoring data. However, the quantitative importance of cigarette butts and their special character justified specific model simulations. When entering the water, cigarette butts face a much faster decay than other plastic items (the reason why OSPAR originally counted it under paper) and change their properties from floating to sinking. As a consequence, it is likely that the retention of cigarette butts during transport in water is higher compared to other plastics.

**Fig. 7:**
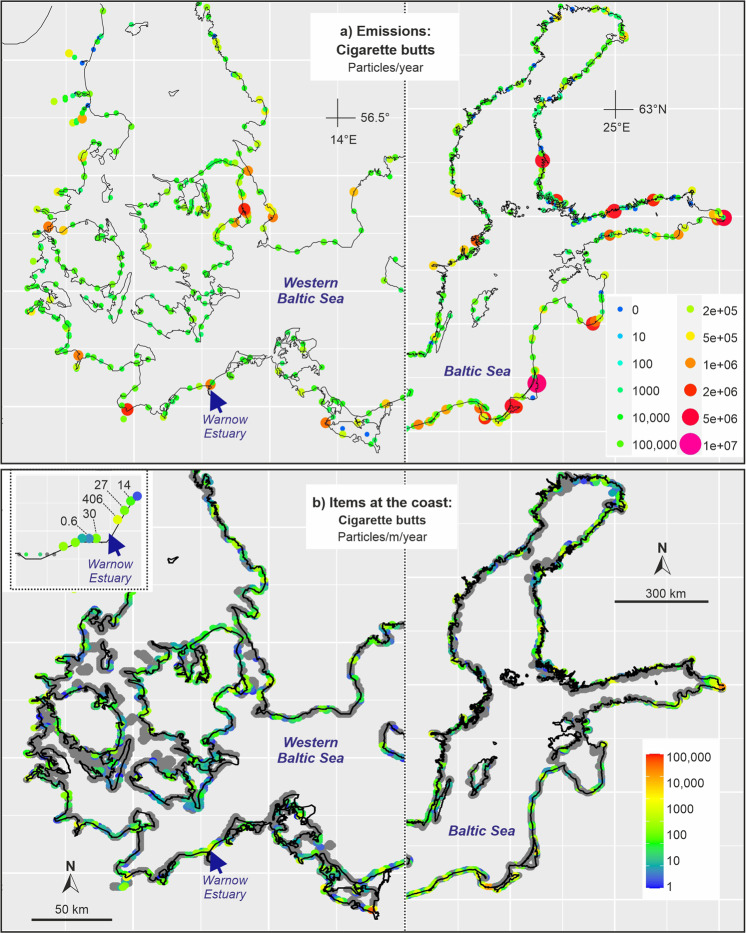
Baltic Sea: **a** emissions of cigarette butts and filters from urban sources (untreated wastewater, stormwater and sewer overflow water) to the Baltic Sea assuming no retention in rivers; **b** accumulation of these items at Baltic Sea shores based on simulations with a 3D hydrodynamic model

**Fig. 8:**
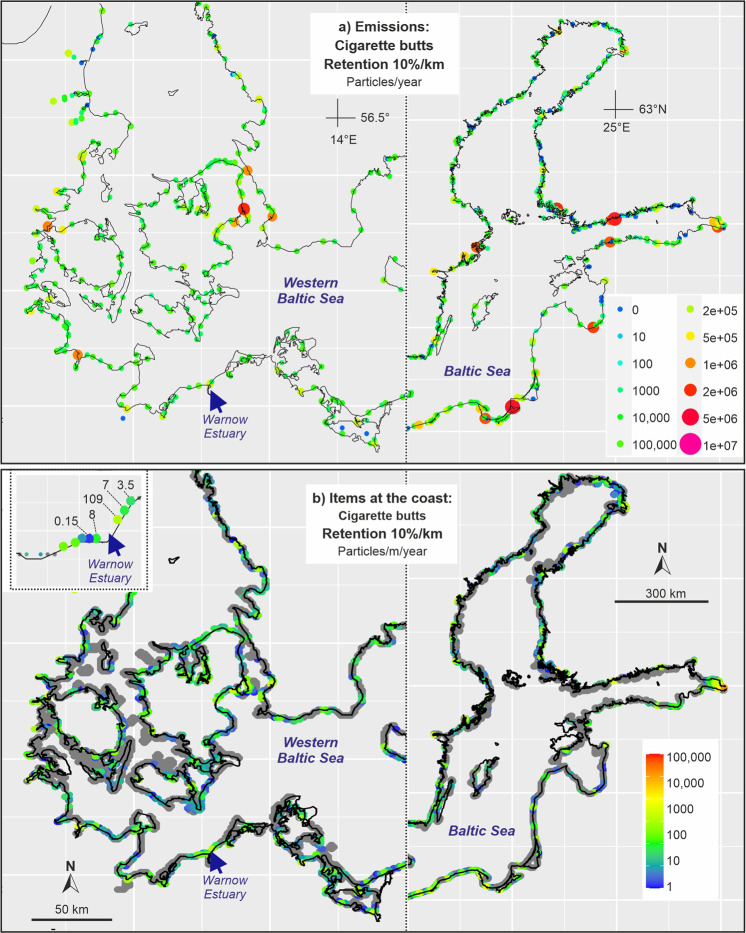
Baltic Sea: **a** emissions of cigarette butts and filters from urban sources (untreated wastewater, stormwater and sewer overflow water) to the Baltic Sea assuming a retention of 10%/km in rivers; **b** accumulation of these items at Baltic Sea shores based on simulations with a 3D hydrodynamic model

Figure 7 shows the emission of cigarette butts to the Baltic Sea and the resulting accumulation at the coastline based on our extrapolated emission calculations. Figure 8 uses the same emission data, but assumed that during the transport in rivers, a retention of the cigarette butts of 10%/km took place. The comparison of Figs. 7a and 8a shows the consequences on emissions to the Baltic Sea. In cases where the emission took place in large coastal cities, such as Helsinki, Stockholm or Copenhagen the difference was minor. Spots where large rivers, such as Daugava (Latvia) and Nemunas (Lithuania), enter the Baltic Sea, the simulation applying a 10% retention/km showed strongly reduced emissions of cigarette butts and subsequently much lower concentrations in the sea and at beaches. Most butts were kept back during the riverine transport.

The assumption of a retention in the Warnow river (and partly in the Warnow Estuary) strongly changed the accumulation at the beaches (Figs. 7b and 8b). Without retention, we got an annual accumulation of 406 cigarette butts/m coastline compared to 109 cigarette butts/m coastline assuming retention of 10%/km. This clearly indicates that the present lack of reliable information about retention of items in the river and the estuary is a serious shortcoming and limits the reliability of our model results.

## Discussion

The model approach and the simulations of emission, transport and deposition of large micro- and mesolitter contain many assumptions and simplifications. For the simulation of microplastics with a size below 500 µm and single plastic types (PE/PP and PET) as well as plastics classes with densities between 0.8 and 1.4 g/cm³, this is discussed in detail in Schernewski et al. (2020, 2021). During these earlier studies, we learnt that differences in plastic particle shape and size do not play an important role for our model simulation results, at least not when taking into account our temporal and spatial model resolution. In our approach, we do not assume that plastic particles (apart from cigarette butts) change their properties during the relatively short time residence of a few weeks in the marine environment. The high share of PP and PE particles in the sediments of the Warnow Estuary indicate that, when the residence time of plastics in the marine environment exceeds weeks, particles can change their buoyancy. This happens, for example, when particles are temporarily trapped in reed belts and organism settlement on plastics takes place. It is likely that the properties of plastics changes towards the properties of natural organic material with a density slightly above the density of water. As a consequence, differences in plastic size, shape and density should play a decreasing role on its behavior in the sea the longer the model simulation period lasts. This would simplify model approaches.

Our approach to calculate the emission of plastic items, such as cigarette butts/filters, cleaning bristles, cotton buds and lolly sticks, for the city of Rostock very much depends on assumptions as well as social and behavioral factors and seems transferable within the Baltic Sea region but not easily beyond. The emissions to the sea further depend on environmental factors such as climate (e.g., likelihood of heavy rains) as well as the type and quality of the urban wastewater system (e.g., combined or separated sewer system). Being aware of the weaknesses we follow an alternative approach to calculate item emissions, based on coastline monitoring within the estuary with a subsequent spatial and temporal extrapolation to get annual emission data. The results of both approaches were consistent and together indicate, that we met the right order of magnitude with respect to our annual item emission data.

The observed plastic concentrations in the Warnow river of 0.32 (200 µm net), 0.016 (300 µm net), and 0.019 plastic particles/m³ (1000 µm net) are low. Applying 300–333 µm nets, Baldwin et al. (2016) report concentrations in Great Lakes tributaries of 0.05–32 particles/m³ and Yonkos et al. (2014) found concentration between 0.27 and 1 particles/m³ in four rivers in the USA. In more urban rivers the concentrations are higher, e.g., 2.4–5.7 particles/m³ (McCormick et al. 2014) and 5 particles/m³ (Mani et al. 2015) or 7 particles/m³ (Faure et al. 2015). Reasons for low concentrations in the Warnow can be the low population density in the catchment and well established sewage treatment systems with a very high connection degree. The concentrations in the estuary of 0.14 particles/m³ (300 µm net) are in the range of existing data. With a similar approach, Setälä et al. (2016) found 0.3–2.1 particles/m³ in the Gulf of Finland and Tamminga et al. (2018) 0.04–0.09 particles/m³ in the South Funen Archipelago. Our model simulated concentrations of large micro- and mesolitter in the water body of the Baltic Sea cannot be sufficiently validated with our field data. To our knowledge, suitable and comparable publications are lacking.

Our data shows that the estuary and nearby Baltic Sea beaches are hot-spots for plastic pollution and accumulation. The highest density of large micro and mesoplastic particles per sampling are observed at Warnemünde (7–31 particles/m²), followed by Hohe Düne (6–17 particles/m²) in flood accumulation zones of beaches. Both beaches are located close to the estuary mouth. At more distant and remote beaches, the numbers are much lower Markgrafenheide (1.2 particles/m²) and Kägsdorf, Nienhagen and Darss (0.3 particles/m²). International literature on macro litter is abundant and shows a wide spectrum of plastic items at Baltic beaches (MARLIN 2013, Schernewski et al. 2018). The same is true for mesolitter (Haseler et al. 2020). However, common beach monitoring methods are hardly suitable to indicate plastics that is washed ashore. For this purpose, the flood accumulation zone method is much more suitable. But it has serious weaknesses, for example, it depends on unpredictable high water level events and can hardly be applied strictly at defined dates, the available time window for carrying out the monitoring after a flood is only a few days, before the accumulation zone is destroyed. For a comparison of methods see (Haseler et al. 2019).

The field data for Baltic Sea beaches and model results are consistent. This is true for the spatial pattern, gradients between locations and the annual absolute amounts of selected items and the meso-plastics item group. However, the absolute annual amounts of visible plastics accumulated at the coast are highly uncertain and depend on the method. Here, we assumed a bi-weekly monitoring in agreement with the EU Water Framework Directive. Another approach would have resulted in different values. Further, we do not know how much of the plastics was already at the beach and was just re-suspended and re-accumulated in the flood edge. The model results depend on the used calculated emission scenarios as model input. Because of these uncertainties we cannot, with respect to the annual amounts, speak of a reliable model validation.

We combine emission calculations, field monitoring in the estuary and at sea beaches as well as modeling but the combination of all data does not allow us to provide reliable information on visible plastics retention in the Warnow river or in the Warnow Estuary. We can assume that, because of a comparable population in the Warnow river catchment, the plastics emissions are similar to our calculations for the city of Rostock, but the low number of particles found in the river mouth monitoring is too weak to allow retention calculations. Data in Piehl et al. (2021) and Labrenz (2020) indicate no permanent microplastics retention in the Warnow river, but make obvious that that transport and processes in rivers are still poorly understood. Our two model simulations on cigarette butts, which assume no retention as well as a retention of 10%/km in rivers, clearly indicate the high importance of retention on items concentrations in the sea and on coastal item accumulation. Hoellein et al. (2019) conclude that current models of microplastic transport underestimate microplastic retention in rivers. This view is supported by results of Besseling et al. (2017) who carried out scenario studies with a hydrological model and conclude that in 40 km river practically all particles (>100 µm spherical polystyrene) are kept back. Our assumed retention of 10%/km for cigarette butts is comparable and means a retention of 98.5% over 40 river kilometers. The problem of retention in rivers with a focus on microplastics emissions to the Baltic Sea is discussed in detail in Schernewski et al. (2021).

The retention of visible plastics in the estuary cannot be quantified, as well. Model approaches are promising, but our spatial model resolution is not sufficient and the data is not consistent. The large amounts of large micro- and mesoplastics found at the estuary shorelines and the abundancy of plastics in estuary sediments clearly indicate that the estuary serves as a sink. However, how much of the emissions, entering with the Warnow river and the city of Rostock, are kept back and how much is exported to the Baltic Sea is uncertain. Piehl et al. (2021) estimate a microplastic retention in the estuary of 50%-90%, but point out the high uncertainty.

Our idea was to use large micro- and mesolitter plastics, which can easily and at low cost be monitored in the field, as an indicator for the plastic pollution in general and to use specific items as indicators for selected pollution sources and/or pathways. We assume stormwater and sewer overflow as major urban pathways for plastics of all size classes in the Baltic Sea region (Schernewski et al. 2020). This is especially true for large micro- and mesolitter. As soon as sewage water passes a Waste Water Treatment Plant, at least 80% of all microplastics is kept back and the retention in modern WWTPs, with at least three treatment stages, is above 95% (Baresel & Olshammar, 2019). Larger plastic fractions are practical fully kept back. Therefore, we focus on stormwater and sewer overflow and considered cleaning bristles, cigarette butts, lolly sticks and cotton buds as suitable indicators for emissions via these pathways. They are easy to monitor in the field, but, apart from cigarette butts, the numbers emitted to the environment are too low that these items can serve as general sewage and stormwater indicators. Cleaning bristles were found only at a few locations. Cotton buds are very specific for this emission pathway but found only in the southern part of the estuary, very close to likely emission spots. Lolly sticks and cigarette butts are not specific enough and can have other pollution sources, e.g., direct emission by tourists. However, cotton butts indicate that sewage overflow are important pollution events in the Warnow Estuary. A weakness is that these items, apart from cigarette butts, are not specified in common beach monitoring protocols. As a consequence, existing beach data does not provide comparable data.

## Conclusions and Recommendations

The Warnow Estuary area, with Rostock city and the Warnow river, is an emission and pollution hot-spots for micro- and mesoplastics. This area can be regarded as typical and representative for several estuaries at the southern Baltic coast and the results seem transferable within the Baltic region. Cities and rivers in the Baltic are important emission hot-spots for visible plastics.

The accumulation of visible plastics at the coastline in the near surrounding of the emission spots is confirmed by our model simulations. Especially in coastal waters, the residence time of plastic items in the water is only days. The short residence time, relatively low plastic concentrations and vertical gradients resulting from different buoyancies, make a sampling in the water body not recommendable. Instead, large micro- and mesoplastics monitoring should focus on final sinks, the beaches. The sampling should catch the spatial gradients from the potential emission hot-spot (e.g., river mouth). For the development of a spatial sampling strategy, spatial high resolved hydrodynamic models are very suitable tools. On beaches, the sampling should focus on the flotsam and be carried out during high water level events. This ensures a sufficient number of particles and that the accumulated items are seaborne. The lack of methods for assessing microplastic fractions below 1 mm at beaches is an existing shortcoming.

The plastics pollution of the Warnow Estuary sediments indicates that sediment studies in areas where organic material accumulates are a suitable complementation to get a full picture of all relevant plastic sinks in coastal systems. Relatively low concentrations of plastics and fluffy organic sediments make the sampling from a boat methodologically difficult. Therefore, we recommend to utilize channel dredging events to assess the plastic composition and abundancy in sediments. However, this requires further methodological developments.

The retention of plastics in rivers (and estuaries) depending on size, form and density is still largely unknown. A better knowledge of emission, retention and loads of visible plastics in rivers is a core requirement for improving the reliability of 3D-hydrodynamic model simulation approaches. Further, much of our plastics accumulated in the estuary sediments was originally floating, but overgrowth with algae and bacteria increased the density and cause a deposition at the bottom. This process seems to play an important, but still insufficiently known, role for the plastic behavior in the marine environment.

Large micro- and meso-plastic emissions from urban sources, especially during sewer overflows and stormwater seem to be of highest importance in the Warnow Estuary. A consequence is that pollution mitigation or reduction measures should preferably focus on urban areas. High emissions can take place during short events, often lasting less than hours. Therefore, the sampling needs to be event-based. However, the quantification of plastic emissions during stormwater events remains a methodological challenge. A general lesson learnt is that traditional sampling programs with defined locations and fixed sampling intervals alone are not sufficient to provide the necessary data for a successful plastics monitoring and management. These approaches need to be complemented by event-based sampling strategies and methods.

The combination of simple beach monitoring with emissions calculation and modeling approaches, in general, are promising for improving our understanding of plastics pollution in time and space. Each of the single applied method has short-comings and uncertainties and the combination of all methods stabilize the results. However, neither large micro- and mesoplastics, as a size class, nor single items are suitable to provide a consistent spatial picture of transport, behavior and deposition of plastics within the estuary and between estuary and the sea or could serve as general pollution indicator for seaborne plastics. A specification of these items in common monitoring protocols, would improve the data basis.

Microplastics emissions in the entire Baltic, too, largely result from wastewater, stormwater and sewer overflow. Neither our selected items nor item classes in the size fraction above 1 mm sufficiently indicate pollution from these pathways. Further, visible plastics in the coastal environment often results from direct and local sources, such as tourism. As a consequence, items or item classes above 1 mm can hardly serve as indicator for the pollution with small microplastics below 1 mm. However, they may serve as an indicator in itself.
